# Expectations From Clinical Attachments Among Undergraduate Nursing Programmes

**DOI:** 10.1002/nop2.70674

**Published:** 2026-08-19

**Authors:** Asefu Awoke Degefa, Henok Biresaw Netsere, Worku Animaw Temesgen

**Affiliations:** ^1^ Department of Adult Health Nursing, School of Health Science, College of Health Science Woldia University Woldia Ethiopia; ^2^ Department of Adult Health Nursing, School of Health Science, College of Medicine and Health Science Bahir Dar University Bahir Dar Ethiopia

## Abstract

**Aim:**

To explore the expectations of undergraduate nursing students from their clinical practice.

**Design:**

A qualitative descriptive study was conducted at Bahir Dar University and University of Gondar, Ethiopia, from 25 April to 25 October 2024.

**Method:**

Representative nursing students were purposively selected for a focus group discussion to explore their expectations in the clinical learning environment. Data were collected using focus group discussion guides. Data were audio‐recorded, verbatim transcribed, translated, coded and analysed using thematic analysis with the help of ATLAS.ti 9.1.

**Results:**

A total of 30 participants (15 male and 15 female) were involved from two public universities with teaching hospitals. Two main themes, including supportive clinical learning environments and learning need expectations, were identified: four subthemes that are suitable clinical setting, supervision and guidance, application of theory into practice and being skillful were developed within each theme.

**Patient or Public Contribution:**

No formal patient or public involvement in this study. Nursing students participated only as research participants.

**Conclusion:**

When students enter clinical settings, they anticipate developing skills, receiving support, being supervised, applying theoretical knowledge to practical situations and experiencing an appropriate clinical learning environment.

**Implications for Nursing Education:**

It requires coordinated efforts amongst policymakers, nursing educators, preceptors and clinical learning sites to enhance the students' motivation, satisfaction and acquisition of nursing knowledge and skills. Policymakers and educators shall revise nursing curricula to better integrate theory and practice. Policymakers should establish national standards and accredit clinical learning environment sites to ensure they provide quality learning opportunities and enhance the overall quality of clinical education. Nurse educators and preceptors shall closely supervise students and limit the number of students assigned per clinical area to avoid overcrowding. Future research shall aim to conduct a mixed study design to explore the depth and breadth of a finding.

**Abbreviations**
BDU: Bahir Dar University
ECCN: Emergency and Critical Care Nursing
FGD: Focus Group Discussion
UOG: University of Gondar

## 1 Introduction

Nursing education consists of two components: the theoretical aspect and clinical learning (Zamanzadeh et al. 2024; Saifan et al. 2021). Students undergo comprehensive theoretical education before putting their knowledge into practice during the clinical component of their course (Ajani and Moez 2011). Clinical learning environments in nursing education offer nursing students the opportunity to apply their theory to practice (Mbakaya et al. 2020; Saifan 2015). When nursing students enter these clinical environments, they may have some expectations regarding an effective clinical learning environment (Ergezen et al. 2022). They have expectations of integrating theory and practice, fitting into the multidisciplinary health team, performing well in both theoretical knowledge and nursing practice and delivering high‐quality nursing care (Mafumo and Netshikweta 2022). All students have expectations regarding what they will learn during their clinical experience (Cowen et al. 2018).

Expectation is defined as the hope or possibility of achieving something (Mafumo and Netshikweta 2022). Expectations, in the context of clinical education, refer to the aspirations and desires of students regarding their learning experiences (Mafumo and Netshikweta 2022; Soler et al. 2021). Students have expressed a sense of belonging as well as increased motivation, commitment, competence, confidence and self‐esteem when their expectations have been met (Taylan and Özkan 2021; Ergezen et al. 2022). It's important to understand the characteristics of good clinical education that meet students' needs and expectations to achieve high‐quality nursing education (Allari and Farag 2017). Understanding students' clinical learning expectations is crucial since this affects their motivation to continue their education and determines their level of satisfaction (Berhe and Gebretensaye 2021; Munangatire et al. 2023; Asefi et al. 2017; Ergezen et al. 2022).

Numerous studies have highlighted the challenges encountered by nursing students and their experiences in the clinical learning environment worldwide, and in Ethiopia (Wawire et al. 2014; Rezakhani Moghaddam et al. 2020; Aryuwat et al. 2024). Most reviewed studies focused on the challenges faced during clinical attachment and some studies showed how they experienced it. However, expectations of nursing students from the clinical practice have not been studied in the Ethiopian context. This absence of data hinders the development of evidence‐based policies and educational practices. Therefore, this study aims to explore the expectations of nursing students from the clinical practice at Bahir Dar University (BDU) and the University of Gondar (UOG).

## 2 Theoretical Framework

This study is guided by Albert Bandura's social cognitive learning theory, which provides a foundational framework for understanding how undergraduate nursing students learn and develop competencies in clinical practice. The theory emphasises that learning occurs through observation, imitation and modelling, which are particularly relevant in clinical settings where students are expected to acquire and apply practical skills. The theory consists of four key phases: attention, retention, reproduction and motivation, which explain the process of learning and skill acquisition (Stanley et al. 2020; Devi et al. 2022; Kilinç et al. 2018). These phases are aligned with the expectations of nursing students in clinical practice.

## 3 Materials and Methods

### 3.1 Study Design

A qualitative descriptive study design was conducted.

### 3.2 Study Setting, Study Participants, Participant Recruitment Process and Study Period

This study was conducted at the College of Medicine and Health Sciences at BDU and UOG, Ethiopia. Both universities are public institutions with teaching hospitals that serve as the main clinical practice sites for nursing students.

The study participants were undergraduate nursing students. Focus group discussions (FGDs) were used for data collection. Students of different genders from the second through the 4th year of university from different nursing programmes (comprehensive nursing, paediatrics, emergency and critical care nursing and psychiatry) were recruited using purposive sampling to ensure sufficient diversity of points of view. Students in their 2nd year and above were purposively selected, since they began their clinical practice in the hospital starting from the 2nd year.

Participants were approached face‐to‐face or by telephone. Participants were continuously recruited from 25 April 2024 to 25 October 2024, throughout the study. First‐year students and 2nd‐year students with less than 1 month of clinical experience were not included because of their limited clinical experience. The aim was to recruit six participants with various nursing programmes (comprehensive nursing, paediatrics, emergency and critical care nursing and psychiatry) per focus group. The number of discussions depended on data saturation. Five FGDs were conducted. Since no new codes or themes emerged in consecutive focus groups, this sample size was considered sufficient. The study was conducted from 25 April to 25 October 2024.

### 3.3 Sampling and Sample Size

The maximum variation purposive sampling method was used to select students based on gender and academic year. Students from each academic year and each specialty/nursing programme were selected. One or two class‐representative students from each specialty/nursing programme were selected for FGDs. Then, students were grouped according to their academic year. Five FGDs were conducted with each group having six participants. FGDs were conducted until the data were saturated.

### 3.4 Data Collection Tool

Data were collected from students through FGD by using the semi‐structured FGD guide with probing techniques, which were adapted from another research (Mafumo and Netshikweta 2022; Soler et al. 2021; Biles et al. 2022).

The discussion guide was structured into three sections: socio‐demographic characteristics, general questions that were designed as icebreakers to stimulate discussion and foster interaction and questions related to nursing students' expectations in clinical practice.

The interview questions related to nursing students' expectations included:

1. Was the clinical placement an effective learning environment? How supportive was the clinical setting for learning?
2. What do you want to be done when you enter clinical attachment? What do you want the instructors and staff to do for you to ensure you have a good learning outcome, achieve competency and improve your performance?
3. What would you like teachers and staff to do to improve your performance and skills?
4. What were your objectives from the clinical practice?
5. Do you believe you attained your objectives from those attachments/clinical practice?
6. At the end of your clinical attachment, what objective or learning outcome do you want to achieve? You have something planned, right? …

Probing questions were posed to participants to enrich and complete the data. Some of the probing questions included, ‘What specifically do you mean by…?’ ‘Can you elaborate on…?’ and ‘Can you explain your reasons further?’ Rephrasing was used to request some explanations; that is, ‘You mentioned that … in the clinical practice; can you tell us more about it?’

The FGD guide was originally prepared in English and then translated back into the local language.

### 3.5 Data Collection Procedure

Before the discussions, participants were informed about the study's purpose, and their permission was obtained. The discussions were conducted in a classroom setting. The principal investigator facilitated the FGD, and a BSC nurse took notes during the discussion. The discussion was recorded with a digital audio recorder. The duration of each FGD was 70 min on average.

### 3.6 Trustworthiness

To ensure the trustworthiness of the study findings, four techniques were used: credibility, dependability, confirmability and transferability (Korstjens and Moser 2018).

*Credibility:* The credibility of this study was maintained through peer debriefing. Peer check was performed by two expert supervisors of nursing and experts in qualitative research, who supervised the process of interviewing, coding and categorisation and reviewed the transcripts, methodology and findings.

*Dependability:* To ensure the dependability of this study, audio recordings of participants' interviews, notes taken during the interviews and verbatim transcriptions were saved for cross‐checking the process and maintaining consistency in the interpretations.

*Transferability (Applicability):* To ensure transferability, the investigators used a thick description of the research context, methodology, participants and final report.

*Confirmability (Neutrality):* To establish confirmability, the researcher used participants' words from transcripts to confirm that the data interpretation reflects the exact participants' words instead of the researcher's perspectives or biases. Moreover, maximum variation of sampling (in terms of gender and academic year) enhanced the confirmability and credibility of the data.

### 3.7 Data Processing and Analysis

Data collection and analysis occurred concurrently for 6 months. The data were analysed using thematic analysis using the ATLAS.ti 9 software. Initially, the recorded data was transcribed into a textual format. The principal investigator read and reread the transcriptions several times and listened to the recorded data repeatedly. Then, the transcripts and audio were cross‐checked several times, and the data were translated into English. Next, initial codes were generated for segments of the data. The codes were allocated into categories. Similar categories were grouped into themes. Finally, the study findings were presented with narrative descriptions and quotes.

### 3.8 Ethical Consideration

The study was conducted according to the guidelines of the Declaration of Helsinki and approved by the institutional review board of Bahir Dar University with protocol number 918/2024. The institutional review board approved the version of informed consent, the date of the informed consent version, the version of the protocol and the date of the protocol version. A letter was written to BDU and the UOG for permission and support. Then, permission was obtained from the department heads of two universities to collect the data from the students. Permission was finally obtained from the instructors assigned to each section in each university to access students. The principal investigator explained to all participants the title of the study, the purpose of the study, the procedure and duration, risks and benefits, confidentiality (personal identifiers, names) and the right to refuse. Then, verbal and written consent were obtained from each study participant. Subsequently, participants signed a written informed consent. At the time of data collection, personal identifiers were excluded. The data were kept strictly confidential and used only for study purposes. All information was kept confidential in a secret place and on password‐protected personal computers.

## 4 Results

### 4.1 Socio‐Demographic Characteristics of Study Participants

A total of 30 participants (15 male and 15 female) were involved from two public universities with teaching hospitals, namely BDU and the UOG. Amongst the study participants, 30 nursing students participated in FGD in five groups.

### 4.2 The Expectations of Nursing Students From the Clinical Practice

#### 4.2.1 Theme One: Supportive Clinical Learning Environments Expectations

This theme has two sub‐themes: suitable/conducive clinical setting and supervision and guidance. The findings are presented below.

##### 4.2.1.1 Subtheme One: Suitable/Conducive Clinical Setting

Students reported that the proximity of the clinical environment to their dormitory and the availability of multiple wards contributed to a positive learning experience.

A 4th‐year nursing student from the paediatrics department described:

*… It is almost a good learning environment. In terms of proximity to our dormitory, it allows us to be available on time. In terms of facilities, the hospital has many wards, and we can see many cases*. (P1, FGD 4)

Nursing students require a clinical learning environment with many cases, which allows them to observe and be involved in nursing care procedures and perform and enhance their skills.

A 2nd‐year nursing student from the ECCN department mentioned:

*I think it is a great place to learn. Since this is a referral hospital, there are many cases to observe and engage with. Being a referral centre often allows us to perform the procedures and tasks necessary for developing our skills*. (P1, FGD 3)

Nursing students often require a clinical learning environment that allows them to observe, experience and participate in various procedures.

A 3rd‐year nursing student in the paediatrics department explained:

*The hospital is somewhat good. The reason why it is good is that, when we compare it with other health centres and primary hospitals, our hospital is a place where many procedures are performed. We found it much better in this regard. If we have the desire to learn, the hospital is the best place because it is specialised and where many procedures are performed. It is where we get good knowledge*. (P5, FGD 2)

To be exposed to diverse cases and to be involved in different procedures, and to observe different procedures, students emphasised the need to attach to the emergency and ICU units.

A 4th‐year nursing student in the comprehensive nursing department described:

… *When we arrive at the hospital, if it could allocate a specific time for the emergency department, similar to how time is allocated for medical, gynaecological, surgical and paediatric rotations, we would gain valuable skills. For instance, it would be beneficial if we had five months of internship, with one month dedicated solely to the emergency department*. (P3, FGD 2)

Another student added:

*We are comprehensive nursing students, which means we need to know everything as nurses. We have to be exposed to all aspects of nursing. However, when we go out on our attachments, the schedule and the wards we are assigned to are not compatible with our learning needs. For example, there are essential skills we need to practice in the emergency department. Every ward we enter is important for our development as nurses. Currently, we are primarily focused on the surgical and medical wards, but we pay very little attention to other areas. We have limited time and no dedicated time for those essential experiences*. (P4, FGD 5)

Another student said:

*…not only in an emergency, but I would like to be exposed in the ICU*. (P3, FGD 1)

Not only in the referral hospital, but students also want to expose themselves in the primary hospital, where cases that did not come to the referral hospital are treated.

A 3rd‐year nursing student in the paediatrics nursing department described:

*……Currently, there are some minor cases that come to this hospital but remain in the primary hospital. Since those cases don't come here and stay at the primary hospital, if I had exposure to them, I would be able to make diagnoses and achieve my goal*. (P1, FGD 1)

Another student from the paediatrics department added:

*If the situation allows, it would be beneficial for us to be attached to the primary hospital. Most paediatric cases are handled at the primary hospital, such as those treated according to the IMNCI* (Integrated Management of Neonatal and Childhood Illness) *guidelines. These cases are left there for treatment, and we are taught based on the IMNCI schedule. It would be advantageous if we had exposure to those cases*. (P5, FGD 2)

Students described the importance of exposing themselves to a variety of cases to learn.

One student said:

*I learn because of exposure. Most people learn more from exposure, not just from what students read or what the teacher taught. Exposure is more useful for us……*. (P3, FGD 2)

Students require a less crowded clinical setting. They reported that too many students assigned to one ward made it difficult to interact with patients and perform procedures.

A 3rd‐year nursing student from a comprehensive nursing department stated:

*When we go to the attachment, the number of students and hospital staff is too high to ask about patients. The number of students assigned to one ward is not proportionate. For example, when we try to gather a patient's history, another student may have already collected that information. As a result, the patient may not provide us the information we seek. When we want to perform a skill, there is also someone else who wants to do it*. (P3, FGD 1)

Students described a situation where the presence of numerous interns and junior students in the same clinical learning environment leads to missed opportunities to acquire basic skills and reduces their ability to practice freely.

A 4th‐year nursing student from a comprehensive nursing department stated that:

*There are interns and juniors, which means there are too many students in one area. From this perspective, we can't acquire the skills we want. It feels very restrictive, and we can't perform procedures freely. Sometimes we think, ‘Since he is a junior, I know more than he does, so let me give him a priority to perform this task’, and we give a chance to perform a task for our juniors, and we end up leaving without practicing. As a result, we lack experience*. (P3, FGD2)

Students reported that health centres, district hospitals and primary hospitals would offer them more opportunities to perform various tasks and procedures than a specialised hospital. This is because too many students in the specialised hospital limited the ability to gain practical experience and active participation in patient care.

A 4th‐year nursing student from an emergency department stated:

*Instead of this specialised hospital, if we are assigned to health centres, district hospitals, or primary hospitals, there will be more opportunities to perform tasks*. (P5, FGD 4)

##### 4.2.1.2 Subtheme Two: Supervision and Guidance

As the students reported, they desire consistent guidance from their instructors and detailed explanations from their instructors in the learning process during clinical practice. Additionally, they want this explanation to extend into the practical application, directly to patients.

This was described by a 2nd‐year nursing student from the paediatrics department:

*We want them* (instructors*) to consistently follow us, explaining everything in detail and helping us to apply what we have learned to patient care*. (P4, FGD 3)

The nursing students desire to be supervised and guided during their clinical attachment. Rather than simply being checked on for attendance, they want the instructors to be more engaged in their daily activities in the hospital setting.

A 4th‐year nursing student from the ECCN department explained this by saying:

*I would be happy if they (instructors) came to the hospital to observe what I was doing and monitor me, and if they said, ‘What are you doing’? I will work hard just by watching them. I think it would be great if they came to the hospital and followed us every day, instead of just coming to take attendance*. (P4, FGD4)

As the students reported, they have a desire to be supervised frequently, and as they stated, this would enhance their knowledge and performance during their clinical attachment. However, they indicated a gap between their expectations and clinical experiences.

A 3rd‐year female nursing student from the paediatrics nursing department mentioned that:

*I thought that there would be daily check‐ups to have better knowledge and performance, and they would ask daily questions like ‘What did you do, what did you encounter, and what hindered your knowledge’, However, this is not the case*. (P2, FGD 1)

Students described that they want experienced instructors or nurses who demonstrate clinical procedures and offer step‐by‐step guidance during their clinical practice. However, their experience was different from what they expected.

A 2nd‐year male nursing student from the paediatrics nursing department described this expectation as follows:

*Our instructors set the expectation that we would see how blood draws are performed when we were in class, just before we entered the hospital for clinical attachment. We anticipated learning about it in the hospital, but when we arrived, there should have been someone to show us how blood draws are performed. If you don't have prior exposure, you need an expert to demonstrate and perform the procedure. When our teacher said, ‘We've seen this in the hospital’, I expected the teacher to guide us through the process step by step and say, ‘Do this, do this, do that, do it like this’. However, when we got there, this guidance was not provided*. (P4, FGD 1)

#### 4.2.2 Theme Two: Learning Needs Expectations

##### 4.2.2.1 Subtheme One: Application of Theory Into Practice in the Clinical Setting

Students reported that they expect to apply their theoretical knowledge in clinical settings. They stated that this would help them integrate their theory into practice.

A 3rd‐year nursing student from the paediatrics nursing department mentioned:

*We looked forward to applying our theoretical knowledge in the clinical area. This was supposed to help us understand how theory and practice come together*. (P2, FGD5)

Students have a desire to be exposed to all theoretical lessons they learn in class and apply or practice all these theoretical concepts during their clinical attachments.

A 3rd‐year male student from the paediatrics department mentioned:

*When we entered the hospital, I believed I would encounter the concepts we learned in theory, such as fundamentals, medical, surgical and psychiatric topics. The teachers had mentioned, ‘We will cover this during the hospital attachment’, and said, ‘You will see this in the hospital’. What I mean is that we expected to see and practice these concepts frequently*. (P1, FGD1)

Based on students' reports, they need to apply the concepts and procedures learned in class directly to practical skills in clinical learning environments. However, they found that the actual experiences in clinical practice differ from what was taught in theory in class.

A third‐year male nursing student from the ECCN department mentioned:

*I expected that the theoretical knowledge I gained in the classroom would be directly applicable in the clinical setting, but I found that the reality was quite different*. (P6, FGD 5)

Students are taught various procedures in their theory. The students desired to apply these procedures consistently in clinical settings. However, they found that these procedures are not consistently observed or practised when they enter clinical settings.

A 2nd‐year female nursing student from the paediatrics department elaborated on their expectations versus experience:

*Whilst we have learned the theory, it has not been fully applied in practice. For instance, even though we have learned procedures, we do not see them applied in practice. We have learned these procedures theoretically, but we do not see them being performed*. (P4, FGD 3)

Another 4th‐year female nursing student from the ECCN department also described their expectations versus reality:

*Most of what we learn in theory is not applied there (hospital)*. (P4, FGD 4)

##### 4.2.2.2 Subtheme Two: Being Competent/Skillful in Practice Expectations

The students described a desire to perform basic nursing skills during their clinical attachment, like medication administration, IV cannula insertion and blood draw.

A 4th‐year female nursing student from the emergency nursing department stated that:

*As a nurse, we know what to do in a clinical attachment. Starting with the basics, I want to be able to perform blood draws perfectly, secure an IV cannula and administer various medications. As an emergency nurse, a lot is expected from me, since I am training to be an emergency nurse. Therefore, I want to be able to do these tasks perfectly*. (P5, FGD 4)

A male student from the comprehensive nursing department also stated:

*I want to learn how to administer medication*. (P4, FGD 2)

Another student mentioned:

*We have learned pharmacology regarding the benefits of medications and their descriptions. However, as nurses, we should also learn how to administer medications properly. For example, we have studied vancomycin antibiotics and their mechanisms of action, but we have not learned how to administer them. This is where we need to develop practical skills. As I mentioned, it would be beneficial if we could adjust our time. Moreover, if teachers could instruct us daily on how to administer medications like acyclovir, vancomycin and KCl, it would greatly enhance our effectiveness in the future*. (P4, FGD 2)

Another student from the paediatrics nursing department added:

*We know the name of the drug; we have learned it, but we don't know how to give it. There was a woman who came after drinking poison. We know what medication to give her; as I told you earlier, we know the name of it. In pharmacology, we know why a medicine is given, and we know the mechanism of action, but we don't know how to administer it. Nurses work on how to administer, not prescribe. There is a very big problem in that part. Because we are nurses, we have to work as nurses. It is administration, not what medicine should be given, like doctors should learn medicine*. (P5, FGD 2)

Most 2nd‐year students enter the hospital for their clinical attachments with the expectation of observing and familiarising themselves with the hospital environment rather than performing tasks. They successfully achieve this expectation.

A 2nd‐year male student from the comprehensive nursing department reported:

*I have achieved my expectations to some extent. The aim of hospital education for us was to observe and understand what we are supposed to do, and to familiarise ourselves with the hospital environment. I believe this expectation has been achieved in terms of observing how things work in the hospital*. (P6, FGD3)

Most 3rd and 4th‐year students enter their hospital attachment with the expectation of performing tasks to develop essential skills. However, despite their strong desire to perform tasks during their clinical attachment, many have found themselves unable to achieve their expectations.

This was described by a 3rd‐year male student from the paediatrics nursing department as follows:

*My answer to the question of whether we have achieved our expectations during the hospital attachment is that I have not achieved my expectations. I am almost finished with my third year now. In my first year, we primarily did observation. At that time, I was already exposed to what was happening in the hospital, and I felt ready for the future. In my second year, it was more about working as an assistant, and we would occasionally assist the clinical staff, but now, since we are nearing the end of our third year, it is time for us to perform the skills on our own. However, I have not been able to do this yet. There are nursing skills to be learned during clinical attachment, but I have not performed any of them so far*. (P1, FGD5)

Another 3rd‐year male student from the paediatrics department also stated that:

*My expectation in paediatrics, since they are children, it is a bit difficult to perform procedures. Right now, I want to practice on them; I mean, I want to develop my skills. What I find difficult is securing an IV cannula in children; even though there are different procedures in paediatrics, usually, securing an IV is considered a skilled task in paediatrics and I believe I can do it. We came into this attachment with that mindset. But I've never tried this. In short, I didn't achieve my expectations. I should have been performing tasks on my own by now. I needed to perform tasks. But I'm not performing it*. (P2, FGD 5)

Beyond performing nursing tasks, the students also have a strong desire to become competent in various nursing tasks during their clinical attachment. They have a desire to master their skills, perform perfectly and gain competency in these tasks.

A 3rd‐year female nursing student from the comprehensive nursing department described:

*I would like to know the skills well. I mean, not only to practice the skills but also to finish my attachment with a competent*. (P4, FGD 1)

Another 3rd‐year female nursing student from the paediatrics nursing department mentioned:

*We wanted to become competent in our skills through hands‐on practice. However, the opportunities to perform certain tasks were limited. What I want to achieve is to perform what a nurse can perform, what a nurse is supposed to do*. (P2, FGD5)

Based on students' reports, they have a desire to be fully capable of performing all the tasks, be able to diagnose and treat minor cases and handle responsibilities that a nurse is expected to handle. They want to reach a level of proficiency where they can handle everything a nurse is supposed to do because they will be responsible for performing nursing tasks independently in their future roles.

A 4th‐year female nursing student from the ECCN nursing department described that:

*What I want to achieve is to perform what a nurse can perform, what a nurse is supposed to do, because the work is waiting for me in the future and I will be alone in the workplace, I will be able to perform my tasks independently, without assistance in the workplace for the future*. (P6, FGD 4)

Another student added:

*…we may have a chance to enter a health centre in the future. Doctors may not be available at the health centre. As a nurse, when a minor case comes in, you have to diagnose, manage and treat it. So, my expectation is to clerk and treat minor cases in the OPD when the patient comes in*. (P4, FGD 2)

Students stated that they need repeated practical experience and multiple opportunities to practice their skills to become competent and build confidence in their clinical skills for future work.

A 4th‐year male nursing student from the comprehensive nursing department stated:

*I would be happy to complete my education in a state where I can work with confidence for the future*. (P2, FGD 4)

All these expectations may not always be fulfilled, and unmet expectations in clinical practices might affect their satisfaction in their study, pursuit of nursing knowledge and skill, motivation to attain required competencies and subsequently their interest in staying in the nursing profession.

## 5 Discussion

The main aim of this study was to explore the expectations of undergraduate nursing students from their clinical practice. Findings from the study show that nursing students have different expectations from their clinical attachments. One of the expectations of nursing students in this study is to desire a supportive/conducive clinical learning environment. That is, they desire a clinical learning environment that has many wards, various cases, is close to their dormitory and is a place where many procedures are performed. This clinical environment allows them to maximize their learning opportunities and gain a good clinical experience, advancing their knowledge and skills.

They also need a less crowded clinical training setting and to be assigned to the health centres, district hospitals, or primary hospitals. Such placement is important to not miss the learning chances of skills, to practice freely and to actively participate in patient care. To gain exposure to a variety of cases, they need to be assigned to the ICU and the emergency department. Although studies from other countries have demonstrated the significance of less crowded and supportive training sites, the findings may not be directly transferable to Ethiopia. Thus, this study offers data specific to the Ethiopian context.

A study conducted in the United Arab Emirates revealed that establishing a supportive clinical environment is vital for enhancing nursing students' engagement in clinical practice, their clinical competencies and satisfaction (Maalouf and El Zaatari 2024).

A study conducted in Italy showed that a well‐organized clinical setting is essential to build confidence, competence and professional identity; support emotional well‐being and motivation; strengthen relationships and teamwork; and facilitate effective, experiential and reflective learning (Lommi et al. 2024). Therefore, healthcare institutions shall prioritize creating and maintaining clinical environments that are rich in learning opportunities. This can be achieved by limiting the number of students assigned per clinical area to avoid overcrowding and facilitating the assignment to several departments so they can learn different cases and procedures. Additionally, policymakers should establish national standards and accredit clinical environment sites to ensure they provide quality learning opportunities and enhance the overall quality of clinical education.

Nursing students in this study also expected supervision and guidance during their clinical practice. They wish to be closely observed in their practice and to receive feedback from their instructor. This close follow‐up from instructors can motivate the students to work harder, and it can help them improve their skills in the clinical setting. Similarly, a study done in South Africa revealed that most learners in the first and second years of the study indicated that when they are in clinical learning areas, they expect professional nurses to offer them support and guide them during the execution of their allocated responsibilities (Mafumo and Netshikweta 2022). This is supported by Bandura's social cognitive learning theory, which is observational learning, where learners acquire new skills and knowledge by observing experienced role models (supervisors) in their environment (Stanley et al. 2020; Kilinç et al. 2018). In the context of this study, nursing students expected supervision and guidance from experienced nurses or clinical instructors, who serve as models for appropriate clinical behaviours (skills, competency). Similarly, a study in Turkey revealed that nursing students expect support and supervision from both the instructor and the clinical staff (Ergezen et al. 2022). Therefore, educators and preceptors consider supervision and mentorship during clinical practice to meet these expectations and increase their competency.

The other expectation of nursing students that was mentioned by students in this study was the application of theory into practice at a real, suitable setting. This study is consistent with other research findings, showing that nursing students expect to integrate both theoretical knowledge and practical experience in their clinical education (Esmaeili et al. 2014; Mafumo and Netshikweta 2022). A study conducted in Sweden found that before their clinical experience, nursing students expected to be able to translate theoretical knowledge into practice and to achieve as much independence as possible in the clinical setting (Liljedahl et al. 2015). Bandura's Social Cognitive Learning Theory, particularly the reproduction stage, reveals that students actively learn by imitating/reproducing the skills and competencies demonstrated by their role models, such as experienced nurses in the clinical setting (Kilinç et al. 2018). This process is essential for skill acquisition. Therefore, this study suggests that clinical supervisors and mentors should demonstrate and provide chances for students to practice these skills during clinical attachments to enhance their competency. Policymakers and educators shall revise nursing curricula to better integrate theory and practice.

This study revealed that nursing students also desired to be competent/skillful in practice. This is because they wanted to be prepared to handle responsibilities independently before they graduated. Similarly, A study conducted in South Africa found that final‐year nursing students expressed a desire to become proficient in performing clinical procedures (Mafumo and Netshikweta 2022). Similarly, A study conducted in Saudi Arabia revealed that most FGD participants expected clinical training to improve their clinical skills, particularly in bedside care, teamwork and the application of evidence‐based practices (Allari and Farag 2017). This is supported by a study conducted in the European Union, which found that nursing students' expectations evolve gradually; by their final year, students anticipate being prepared to become independent and competent practitioners. They also desire mentors who will guide them through the remainder of their training (Lovrić et al. 2017). This aligns with a study conducted in the United States, where students were asked about their expectations for learning in the clinical environment. Most of their responses focused on acquiring nursing skills and procedures (Cowen et al. 2018). Therefore, this study suggests that clinical staff should provide the opportunity to practice and develop skills for the students. Policymakers and educators shall revise nursing curricula to better incorporate the nursing content (e.g., medication administration in pharmacology) that is important for their practice and skills.

## 6 Conclusions

Nursing students enter clinical learning environments with the expectations of integrating theoretical knowledge into practice, developing competence in their skills and receiving support and guidance. These expectations may not always be fulfilled, and unmet expectations in clinical practices might affect their satisfaction in their study, pursuit of nursing knowledge and skill, motivation to attain required competencies, and subsequently, their interest in staying in the nursing profession. On the other hand, meeting these expectations positively impacts clinical learning outcomes, competency and the nursing profession. To meet these expectations, it is crucial to provide support, create an environment with good learning opportunities, closely supervise students and allow students to practise their skills during clinical attachments. Future research shall aim to conduct both an in‐depth exploration and a breadth of all aspects of the findings.

## 7 Limitations

This study was conducted with a relatively small sample, which may limit the transferability of the findings. Additionally, the study might not have explored all aspects. This is a qualitative study, so potential researcher bias in data collection and interpretation may shape the study's findings. Additionally, the cultural and institutional contexts of the two universities may have influenced participants' responses. Future research could strengthen and extend these findings by including a larger sample size, more diverse institutional and cultural settings and mixed‐method designs to explore the depth and breadth of all aspects of the findings.

## Author Contributions

**Asefu Awoke Degefa:** conceptualization, data curation, methodology, formal analysis, writing – original draft. **Henok Biresaw Netsere:** supervision, methodology, validation of data, writing, review and editing. **Worku Animaw Temesgen:** conceptualization, methodology, supervision, writing – review and editing, validation of data.

## Funding

No funding was received for this study.

## Ethics Statement

The study was conducted according to the guidelines of the Declaration of Helsinki and approved by the institutional review board of Bahir Dar University with protocol number 918/2024. The institutional review board approved the version of informed consent, the date of the informed consent version, the version of the protocol and the date of the protocol version. A letter was written to BDU and the UOG for permission and support. Then, permission was obtained from the department heads of two universities to collect the data from the students. Permission was finally obtained from the instructors assigned to each section in each university to access students. The principal investigator explained to all participants the title of the study, the purpose of the study, the procedure and duration, risks and benefits, confidentiality (personal identifiers, names) and the right to refuse. Then, verbal and written consent obtained from each study participant. Subsequently, participants signed a written informed consent. At the time of data collection, personal identifiers were excluded. The data were kept strictly confidential and used only for study purposes. All information was kept confidential in a secret place and on password‐protected personal computers.

## Conflicts of Interest

The authors declare no conflicts of interest.

## Data Availability

The data that support the findings of this study are available on request from the corresponding author. The data are not publicly available due to privacy or ethical restrictions.
